# Major Histocompatibility Complex Class I and Dengue Hemorrhagic Fever: A Meta-Analysis of Human Leukocyte Antigens A*24 and B*44

**DOI:** 10.7759/cureus.31485

**Published:** 2022-11-14

**Authors:** Andrew C Cook, Dylan Thibaut, Teresa Pettersen

**Affiliations:** 1 Medicine, Lake Erie College of Osteopathic Medicine (LECOM), Bradenton, USA

**Keywords:** mhc class i, hla-a*24, hla-b*44, dengue fever, dengue hemorrhagic fever

## Abstract

Introduction: Dengue fever (DF) is a disease caused by dengue virus (DENV) from the family Flaviviridae. The role of human leukocyte antigens (HLAs) in dengue fever (DF) and its more severe manifestation, dengue hemorrhagic fever (DHF), has been a topic of great research interest. In addition to HLA profile, race, age, DENV serotype, infection while having certain chronic diseases, and secondary infection are risk factors for DHF susceptibility. Antibody-dependent enhancement (ADE) of dengue virus infection is a mechanism for DHF infection. Individual studies have examined the effects of HLA-A*24 and HLA-B*44 presence on DHF, but none have analyzed these in a meta-analysis. The objective of this study was to determine the effects of HLA-A*24 and HLA-B*44 presence on DHF and DF susceptibility.

Materials and methods: A meta-analysis on DHF and DF susceptibility in patients with HLA-A*24 and HLA-B*44 was conducted. Google Scholar was used to find studies that contained patients with HLA-A*24 or HLA-B*44 that were diagnosed with DHF or DF. Studies containing patients diagnosed using the 1997 WHO guidelines and possessing HLA-A*24 or HLA-B*44 that were diagnosed with DHF or DF, including primary or secondary infection, and studies measuring odds ratios (ORs) were included. Patients diagnosed using the 2009 WHO guidelines and studies in a foreign language, using animals, or lacking odds ratios were excluded. The National Institutes of Health (NIH) quality assessment of the case-control study tool was used, and a Doi plot was generated using MetaXL to assess for risk of bias. Review Manager version 5.4 was used to generate odds ratios and forest plots with subgroup analysis from allele and phenotype frequency data. Ten studies from 2001 to 2015 met the inclusion criteria. The studies included 2837 DHF/DF patients and 4880 healthy control (HC) patients.

Results: HLA-A*24 was associated with a 1.39 times susceptibility to DHF while those possessing HLA-B*44 were 0.62 times susceptible to DHF (OR=1.39 and 95% CI=1.17-1.66; OR=0.62 and 95% CI=0.39-0.99). Neither HLA-A*24 nor HLA-B*44 presence was associated with DF susceptibility (OR=1.04 and 95% CI=0.82-1.33; OR=0.88 and 95% CI=0.68-1.14).

Conclusion: These results indicate that two different major histocompatibility complex (MHC) class I alleles, HLA-A*24 and HLA-B*44, have opposing effects on DHF susceptibility but none on DF susceptibility. The study's specificity is limited in that it examines HLA allele groups and not specific HLA proteins. The results of this study can be used clinically to identify patients that may be at a higher risk of developing DHF based on their HLA profile.

## Introduction

Dengue fever (DF) is a disease caused by dengue virus (DENV) from four different possible serotypes: DENV-1, DENV-2, DENV-3, or DENV-4 [1]. Dengue virus is a member of the family Flaviviridae,which are positive-strand RNA viruses [1]. Other members of this family include West Nile virus, Japanese encephalitis virus, St. Louis encephalitis virus, zika virus, hepatitis C virus, and yellow fever virus [1]. Dengue fever affects 100-400 million individuals per year globally, with 70% of cases from Asia [1]. However, it affects countries from the Americas, Western Pacific, Africa, and Eastern Mediterranean [1]. *Aedes aegypti* and *Aedes albopictus* are the vectors for dengue virus, which transmit it to humans upon biting the subject [1].

According to the 1997 WHO classification guidelines for dengue clinical diagnosis, symptomatic infections are subdivided into undifferentiated fever, dengue fever (DF) syndrome, or dengue hemorrhagic fever (DHF) [2]. Eighty percent of dengue virus infections are asymptomatic [1]. DF is the mild presentation of the disease and can be characterized as without hemorrhage or with hemorrhage [2]. At least two of the following symptoms are required for diagnosis: leukopenia, hemorrhage, rash, arthralgia, headache, retro-orbital pain, or myalgias [2]. However, some individuals develop the more clinically severe disease, DHF [2]. DHF diagnosis requires the following symptoms: plasma leakage, high fever, hemorrhagic phenomena, thrombocytopenia with hemoconcentration, or circulatory failure [2]. DHF can be further subdivided into without shock or dengue shock syndrome (DSS) [2]. DSS is a more severe form of DHF resulting in circulatory failure, hypovolemic shock, lethargy, or acute abdominal pain [2]. The diagnosis of DSS requires all signs of DHF plus shock [2]. In 2009, the WHO updated the dengue fever classification system [3]. Dengue fever is classified as non-severe or severe [3]. Severe dengue is determined by plasma leakage, severe bleeding, and severe organ impairment [3]. Non-severe dengue is further classified with or without warning signs [3]. These warning signs include abdominal pain, persistent vomiting, fluid accumulation, mucosal bleeding, lethargy, hepatomegaly, or increased hematocrit with a decrease in platelet count [3]. These guidelines were updated to more practically guide clinicians in the diagnosis of a patient with severe dengue without the strict parameters of the 1997 protocol [3]. However, researchers continue to use both classifications.

Human leukocyte antigens (HLAs) are the genes that encode major histocompatibility complexes (MHCs) [4]. MHCs are proteins that are involved in the adaptive immune system [4]. MHC class I is involved in intracellular antigen presentation to cytotoxic cluster of differentiation 8+ (CD8+) T cells [4]. MHC class II is involved in the presentation of extracellular antigens leading to the activation of cluster of differentiation 4+ (CD4+) T cells [4]. Much research has investigated the role of MHC class I and II with susceptibility and severity of dengue fever [5-14]. For MHC class I, HLA-A and HLA-B have been an area of focus for dengue severity research [5-14]. For MHC class II, HLA-DRB1 and HLA-DQB1 have been investigated [5-9].

In addition to HLA profile, DHF susceptibility is increased with secondary infection [15] and DENV-2 serotype [15], in young infants and the elderly [16], in pregnant patients [17], and in patients with bronchial asthma, diabetes mellitus, and sickle cell anemia [18]. DHF susceptibility is also affected by race; African descent was shown to decrease the risk of DHF infection [19].

Antibody-dependent enhancement (ADE) is the mechanism by which dengue virus infection can lead to the more severe disease state, DHF [20]. This is due to the dengue virus attaching to the dengue antibody, which is incapable of neutralizing the virus [20]. As a result, the active virus is internalized into the cell via the Fc gamma receptors (FcgammaRs) [20]. This leads to an increased dengue viremia and worse clinical manifestations such as DHF [20]. 

In this study, we conducted a meta-analysis of the MHC class I allele groups, HLA-A*24 and HLA-B*44, in its role in DHF susceptibility. MHC class I plays a role in viral immunity [21]. HLA-A*24 and HLA-B*44 are frequently mentioned in the literature and warrant further research in a meta-analysis to determine their role in dengue immunity. For example, in a Vietnamese population, Nguyen et al. found HLA-A*24 presence to lead to DHF susceptibility [7]. For HLA-B*44, Vejbaesya et al. found it to be protective against DHF [13].

## Materials and methods

**Figure 1:**
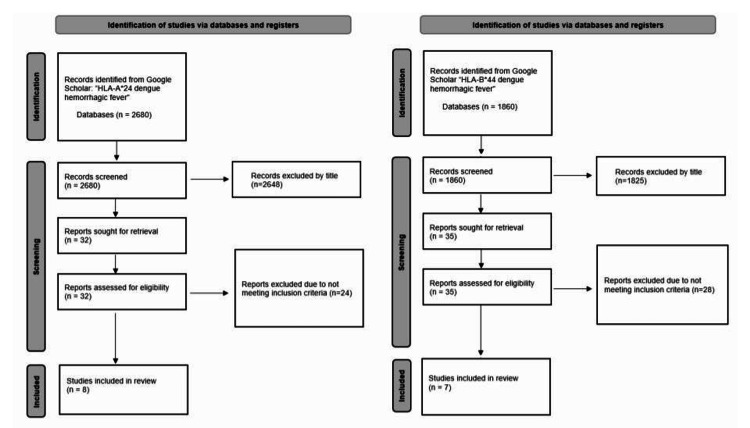
PRISMA 2020 flow diagrams for HLA-A*24 and HLA-B*44 studies HLA-A*24 is represented on the left, and HLA-B*44 is represented on the right HLA: human leukocyte antigen; PRISMA: Preferred Reporting Items for Systematic Reviews and Meta-Analyses

A meta-analysis of DF and DHF patients with HLA-A*24 and HLA-B*44 was conducted in accordance with the Preferred Reporting Items for Systematic Reviews and Meta-Analyses (PRISMA) guidelines. The following inclusion criteria were used: patients possessing HLA-A*24 or HLA-B*44 that were diagnosed with DHF or DF, including primary or secondary infection; studies using odds ratio (OR) or sufficient data available to calculate an odds ratio; and studies using the 1997 WHO guidelines [2]. The following exclusion criteria were implemented: studies including patients diagnosed with dengue shock syndrome (DSS), patients diagnosed according to the 2009 WHO guidelines [3] of severe dengue and non-severe dengue, studies in a foreign language, studies using animals as subjects, and studies not using odds ratio or studies with insufficient data available to calculate odds ratios. The 1997 guidelines [2] were used to encompass all studies within the full publication date range. The 2009 guidelines [3] were excluded to avoid confounding the two guidelines. Google Scholar database was used in the literature review search. The following search terms were used: "HLA-A*24 Dengue Hemorrhagic fever" and "HLA-B*44 Dengue Hemorrhagic fever" (Figure 1). Limitations on study publication date from 2000 to 2022 will be applied. The protocol was published prior to the research on May 20, 2022 [22]. Version 2 was published on May 29, 2022 [22]. The protocol correction was implemented to note the collection and analysis of phenotype frequencies in addition to allele frequencies in this research, as well as clarification on the use of sensitivity testing [16].

Two independent reviewers selected studies in this research according to the following criteria. If a decision could not be reached by the two reviewers, the principal investigator (PI) made the final decision. Data was extracted from figures in the articles. This data included ORs with 95% CIs if directly provided. If this was not directly provided, allele frequencies or phenotype frequencies were collected to generate ORs and 95% CIs. This data was used to determine the odds of DF and DHF given the presence of HLA-A*24 or HLA-B*44.

**Figure 2:**
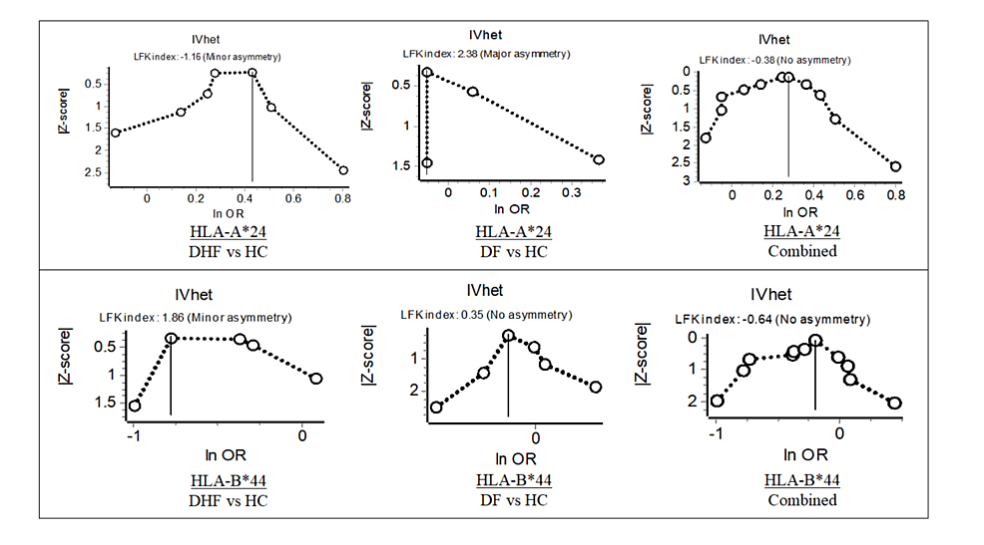
Doi plots for HLA-A*24 and HLA-B*44 in DHF versus HC, DF versus HC, and combined DF: dengue fever, DHF: dengue hemorrhagic fever, HC: healthy control; HLA: human leukocyte antigen

Risk bias assessment was conducted using the NIH quality assessment of case-control study tool, and a Doi plot was generated (Table 1 and Figure 2). Two reviewers independently assessed the studies for bias. If a study met all the selection criteria, two independent reviewers extracted HLA-A*24 and HLA-B*44; allele frequency/phenotype frequency data among those with dengue hemorrhagic fever will be compared to those with the given allele who are healthy. This was repeated with dengue fever as well. This data was inputted into Review Manager version 5.4 to generate odds ratios from each study [23]. Odds ratio was used to measure effect size. These odds ratios were used to create forest plots. If a study contained multiple populations, these were combined into one odds ratio. I^2^ was used in consideration for heterogeneity. MetaXL software was used for sensitivity testing.

**Table 1:** NIH quality assessment of case-control study tool for risk of bias assessment NIH: National Institutes of Health

	Nguyen et al.	Vejbaesya et al.	Loke et al.	Stephens et al.	Alagarasu et al.	Brown et al.	Polizel et al.	Falcón-Lezama et al.	Malavige et al.	Appanna et al.
1. Was the research question or objective in this paper clearly stated and appropriate?	Yes	Yes	Yes	Yes	Yes	Yes	Yes	Yes	Yes	Yes
2. Was the study population clearly specified and defined?	Yes	Yes	Yes	Yes	Yes	Yes	Yes	Yes	Yes	Yes
3. Did the authors include a sample size justification?	Yes	Yes	Yes	Yes	Yes	Yes	Yes	Yes	Yes	Yes
4. Were controls selected or recruited from the same or similar population that gave rise to the cases (including the same timeframe)?	Yes	Yes	Yes	Yes	Yes	Yes	Yes	Yes	Yes	Yes
5. Were the definitions, inclusion and exclusion criteria, algorithms, or processes used to identify or select cases and controls valid, reliable, and implemented consistently across all study participants?	Yes	Yes	Yes	Yes	Yes	Yes	Yes	Yes	Yes	Yes
6. Were the cases clearly defined and differentiated from controls?	Yes	Yes	Yes	Yes	Yes	Yes	Yes	Yes	Yes	Yes
7. If less than 100% of eligible cases and/or controls were selected for the study, were the cases and/or controls randomly selected from those eligible?	Yes	Yes	Yes	Yes	Yes	Yes	Yes	Yes	Yes	Yes
8. Was there use of concurrent controls?	Yes	Yes	Yes	Yes	No	Yes	Yes	Yes	Yes	Yes
9. Were the investigators able to confirm that the exposure/risk occurred prior to the development of the condition or event that defined a participant as a case?	Yes	Yes	Yes	Yes	Yes	Yes	Yes	Yes	Yes	Yes
10. Were the measures of exposure/risk clearly defined, valid, reliable, and implemented consistently (including the same time period) across all study participants?	Yes	Yes	Yes	Yes	Yes	Yes	Yes	Yes	Yes	Yes
11. Were the assessors of exposure/risk blinded to the case or control status of participants?	Yes	Yes	Yes	Yes	Yes	No	No	Yes	No	Yes
12. Were key potential confounding variables measured and adjusted statistically in the analyses? If matching was used, did the investigators account for matching during study analysis?	No	Yes	No	Yes	Yes	Yes	Yes	Yes	Yes	Yes

## Results

A total of 10 studies met the inclusion criteria for the HLA-A*24 and HLA-B*44 analyses [5-14,17]. Eight studies met the inclusion criteria for the HLA-A*24 analysis [7-14]. The studies were published between 2001 and 2015 [7-14]. The studies included Thai, Brazilian, Western Indian, Sri Lankan, Malaysian, and Vietnamese populations [7-14]. The DF versus HC analysis included four studies [9,12-14]. The DHF versus healthy control (HC) analysis included seven studies [1,8,10-14]. The DF studies included a total of 575 DF patients and 1144 HC patients, 188 DF+HLA-A*24 patients, and 278 HC+HLA-A*24 patients [9,12-14]. The DHF studies included a total of 1065 DHF patients and 1648 HC patients, 351 DHF+HLA-A*24 patients, and 438 HC+HLA-A*24 patients [1,8,10-14]. In total, there were 539 patients diagnosed with DHF/DF+HLA-A*24 and 716 with HC+HLA-A*24, 1640 DF/DHF patients, and 2792 HC patients [7-14].

Seven studies met the inclusion criteria for the HLA-B*44 analysis [5,6,8,9,12-14]. The studies were published between 2002 and 2015 [5,6,8,9,12-14]. The studies included Thai, Brazilian, Mexican Mestizo, Jamaican, and Western Indian populations [5,6,8,9,12-14]. The DF versus HC analysis included six studies [5,6,9,12-14]. The DHF versus HC analysis included five studies [6,8,12-14]. The DF studies included a total of 669 DF patients and 1339 HC patients, 161 DF+HLA-B*44 patients, and 282 HC+HLA-B*44 patients [5,6,8,9,12-14]. The DHF studies included a total of 528 DHF patients and 749 HC patients, 78 DHF+HLA-B*44 patients, and 170 HC+HLA-B*44 patients [6,8,12-14]. In total, there were 239 patients diagnosed with DHF/DF+HLA-A*24 and 452 with HC+HLA-A*24, 1197 DF/DHF patients, and 2088 HC patients [5,6,8,9,12-14].

**Figure 3:**
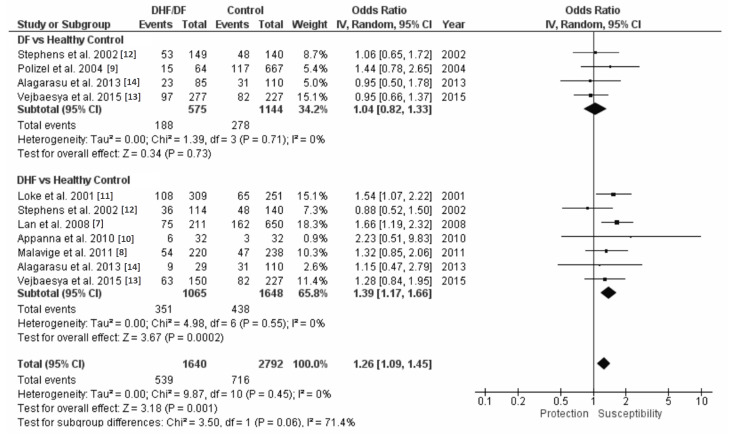
HLA-A*24 forest plots with subgroup analysis of DF versus healthy control (HC) and DHF versus HC HLA: human leukocyte antigen; DF: dengue fever; DHF: dengue hemorrhagic fever

**Figure 4:**
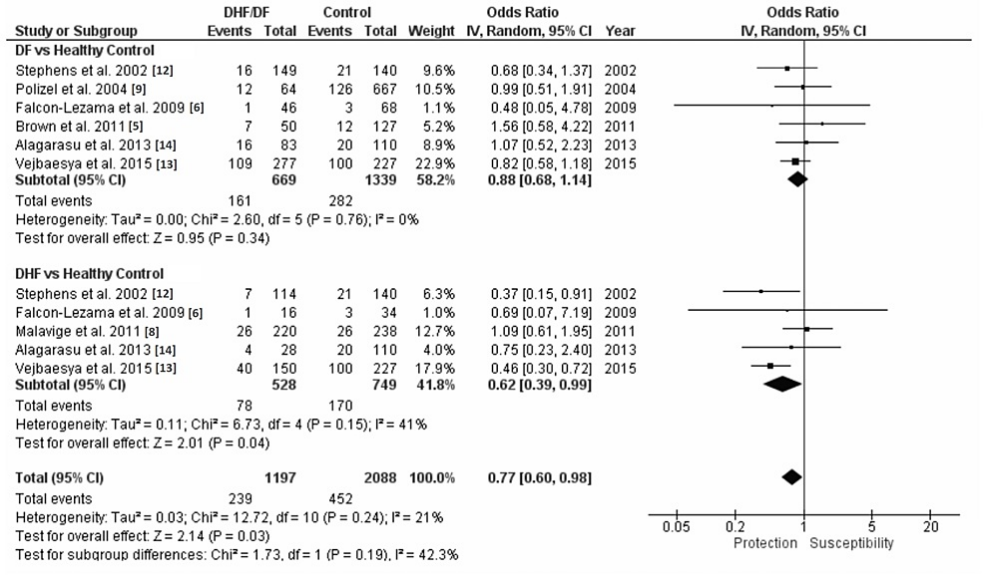
HLA-B*44 forest plots with subgroup analysis of DF versus HC and DHF versus HC HLA: human leukocyte antigen; DF: dengue fever; DHF: dengue hemorrhagic fever; HC: healthy control

A combined odds ratio and 95% CI with subgroup analysis of DF versus HC and DHF versus HC for each HLA examined in this study was generated using the inverse variance (IV) random effect model. Review Manager version 5.4 was used in the generation of these analyses. Figure 3 shows that in the DF versus HC with HLA-A*24 analysis, HLA-A*24 is not associated with susceptibility or protection to DF (OR=1.04 and 95% CI=0.82-1.33). The DHF versus HC with HLA-A*24 analysis shows that HLA-A*24 is associated with a 1.39 times susceptibility to DHF (OR=1.39 and 95% CI=1.17-1.66) (Figure 3). The DF/DHF versus HC with HLA-A*24 analyses found a 1.26 times susceptibility to DF/DHF (OR=1.26 and 95% CI=1.09-1.45) (Figure 3). Figure 4 shows that in the DF versus HC with HLA-B*44 analysis, HLA-B*44 is not associated with susceptibility or protection to DF (OR=0.88 and 95% CI=0.68-1.14). The DHF versus HC with HLA-B*44 analysis shows that HLA-B*44 is protective to DHF with a 0.62 times susceptibility to DHF (OR=0.62 and 95% CI=0.39-0.99) (Figure 4). The DF/DHF versus HC with HLA-B*44 analysis shows a protective effect with a 0.77 susceptibility to DF/DHF (OR=0.77 and 95% CI=0.60-0.98) (Figure 4).

Doi plots were generated for individual subgroups, as well as overall combined groups for both HLA-A*24 and HLA-B*44 (Figure 2). Although the individual HLA-A*24 Doi plots showed minor (hemorrhagic fever versus healthy controls) and major (dengue versus healthy controls) asymmetry, combined subgroup analysis indicated a Luis Furuya-Kanamori (LFK) index of -0.38 (Figure 2). HLA-B*44 Doi plots showed minor asymmetry (hemorrhagic fever versus healthy controls) and no asymmetry (dengue versus healthy controls); there was a combined subgroup analysis Doi plot indicating no asymmetry with an LFK index of -0.64 (Figure 2).

## Discussion

The results of this meta-analysis indicate differing effects of HLA-A*24 and HLA-B*44 presence on DHF susceptibility but not DF. HLA-A*24 presence led to a 1.39 times susceptibility to DHF as compared to HC (OR=1.39 and 95% CI=1.17-1.66). HLA-B*44 had a protective effect from DHF as indicated by the 0.62 times susceptibility to DHF as compared to HC (OR=0.62 and 95% CI=0.39-0.99). Both HLA-A*24 and HLA-B*44 presence did not have a protective or increased susceptibility effect on DF infection compared to HC (OR=1.04 and 95% CI=0.82-1.33; OR=0.88 and 95% CI=0.68-1.14).

The studies used in this meta-analysis were subject to the following limitations. The studies used only contained specificity up to the HLA allele group rather than the specific HLA protein. These factors limit the study in its specificity of HLA-A*24 and HLA-B*44 on DHF. An additional limitation of this study includes potential disease heterogeneity. Future studies should examine HLA associations with DHF in additional different populations. This could investigate whether certain populations are associated with certain HLA profiles for DHF susceptibility compared to others.

In a Vietnamese population, Loke et al. showed a significant effect of HLA-A*24 presence on DHF susceptibility (P<0.05) (OR=1.54 and 95% CI=1.05-2.25) [11]. Additionally, they found a significant protective effect of HLA-A*33 to DHF (P<0.05) (OR=0.56 and 95% CI=0.34-0.93) [11]. In a Sri Lankan population at a Colombo hospital, Malavige et al. found that HLA-A*24 presence was significantly associated with primary DHF infection (P<0.05) [8]. However, there was a nonsignificant effect of HLA-A*24 on DHF susceptibility against healthy control (OR=1.32 and 95% CI=0.85-2.06) [8]. In a Jamaican population, Brown et al. found a significant positive association of HLA-A*24 with dengue infection versus HC (P<0.05) (RR=11.5) [5]. Nguyen et al. found in a Vietnamese population that HLA-A*24 had a positive association with DHF (OR=1.66 and 95% CI=1.19-2.32) [7]. They also found specifically that HLA-A*24 with histidine at codon 70 (A*2402/03/10) was higher in DHF versus HC (HCMC 02-03 DHF: OR=1.75; VL 02-03: OR=1.46; VL 04-05: OR=2.02, P<0.05) [7].

Vejbaesya et al. found in a Thai population that HLA-B*44 was found to have a significant protective effect against secondary DHF infection (P<0.05) (OR=0.46 and 95% CI=0.30-0.72) [13]. In a different Thai population, Stephens et al. also found HLA-B*44 to have a significant protective effect against secondary DHF infection; however, after the p-value was corrected, it was no longer significant (OR=0.37 and 95% CI=0.15-0.91) [12]. However, Xavier Eurico de Alencar et al. found a significant increased susceptibility to DHF compared to DF after DENV-3 infection in patients with HLA-B*44 (P<0.05) (OR=2.025 and 95% CI=0.97-4.24) [24].

HLA-A*24 and HLA-B*44 are among the most frequently examined in the literature [5-7,9,12-14]. However, different studies that are limited to certain populations have found significant associations with other class I HLAs [5,8,12-14]. Brown et al. found in a Jamaican population that HLA-A*23 was associated with a significant protection to dengue infection (P<0.05) (RR=0.05) [5]. Malavige et al. found a significant positive association of HLA-A*31 with DSS during dengue infection in a Sri Lankan population (P<0.05) (OR=18.58 and 95% CI=2.185-158.0) [8]. In a Thai population, Stephens et al. found significant positive associations of HLA-A*2 in secondary DF, DHF, and combined versus controls, of which the DF and combined were significant after statistical correction (P<0.05) (OR=2.58 and 95% CI=1.49-4.50; OR=2.10 and 95% CI=1.20-3.66; OR=2.33 and 95% CI=1.46-3.72) [12]. In a Western Indian population, Alagarasu et al. found that HLA-A*02:11 was positively associated with DHF versus DF (OR=2.29 and 95% CI=0.84-6.09) [14]. In addition, they found that HLA-A*33 was positively associated with DF versus HC (OR=2.12 and 95% CI=0.99-4.52) [14]. Vejbaesya et al. found in a Thai population that HLA-A*02 and HLA-A*01/03 had a positive association with secondary dengue infection (OR = 1.92 and 95% CI=1.30-2.83; OR=3.01 and 95% CI=1.01-8.92) [13].

Stephens et al. found a significant association, including after correction, with HLA-B*51 and secondary DHF versus HC in a Thai population (P<0.05) (OR=4.11 and 95% CI=1.44-12.28) [12]. In a Mexican Mestizo population, Falcón-Lezama et al. found a statistically significant protective association, including after statistical correction, of HLA-B*35 in DF/DHF versus HC and DF versus HC (P<0.05) (OR=0.12 and 95% CI=0.037-0.39; OR=0.13 and 95% CI=0.031-0.51) [6]. Alagarasu et al. found in a Western Indian population that HLA-B*18 was significantly positively associated with both combined DHF/DF and DF infection versus HC (P<0.05) (OR=3.53 and 95% CI=0.87-20.43; OR=2.12 and 95% CI=0.99-4.52) [14]. Appanna et al. found in a Malaysian population that HLA-B*13 had a significant increase in susceptibility to DHF versus HC (P<0.05) (OR=0.18 and 95% CI=0.03-0.90) [10].

## Conclusions

This study showed that two different MHC class I allele groups, HLA-A*24 and HLA-B*44, had opposing effects on DHF susceptibility but no effect on DF. HLA-A*24 presence leads to an increase in DHF susceptibility, whereas HLA-B*44 had a protective effect. More data should be collected on isolating the specific HLA alleles involved among the HLA-A*24 and HLA-B*44 allele groups. Then, once this data is available, further research should be conducted to determine which specific HLA allele is involved in DHF susceptibility. This research can be clinically significant in identifying a patient's risk based on their HLA profile to DHF.
